# Tuning the Electronic Properties of Graphane via Hydroxylation: An Ab Initio Study

**DOI:** 10.1021/acs.jpcc.1c04397

**Published:** 2021-07-20

**Authors:** Francesco Buonocore, Andrea Capasso, Massimo Celino, Nicola Lisi, Olivia Pulci

**Affiliations:** † ENEA, Casaccia Research Centre, I-00123 Rome, Italy; ‡ International Iberian Nanotechnology Laboratory, 4715-330 Braga, Portugal; § Department of Physics, and INFN, University of Rome Tor Vergata, I-00133 Rome, Italy

## Abstract

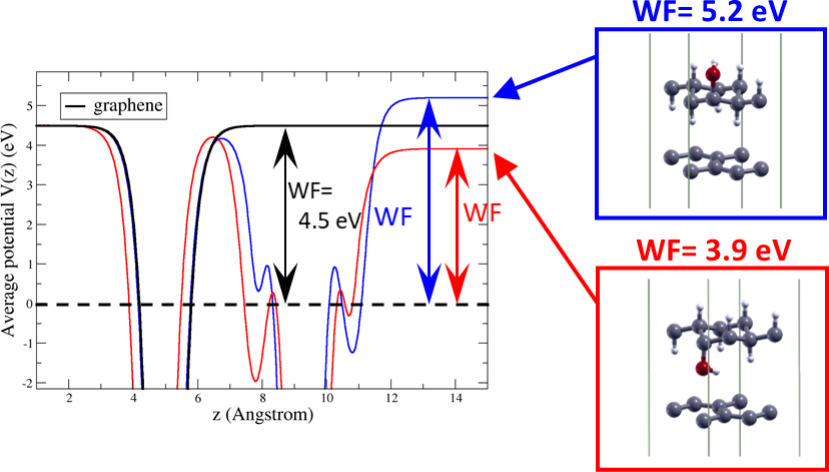

The thermodynamic stability of hydroxylated graphane, that is, fully sp^3^ graphene derivatives coordinated with −H and −OH groups, has been recently demonstrated by ab initio calculations. Within the density functional theory approach, we investigate the electronic property modifications of graphane by progressive hydroxylation, that is, by progressively substituting −H with −OH groups. When 50% of graphane is hydroxylated, the energy bandgap reaches its largest value of 6.68 eV. The electronic affinity of 0.8 eV for graphane can widely change in the 0.28–1.60 eV range depending on the geometric configuration. Hydroxylated graphane has two interfaces with vacuum, hence its electron affinity can be different on each interface with the formation of an intrinsic dipole perpendicular to the monolayer. We envisage the possibility of using hydroxylated graphane allotropes with tunable electronic affinity to serve as interfacial layers in 2D material-based heterojunctions.

## 1 Introduction

Chemical modifications of graphene are able to extend the properties of the pristine material and unlock features of great interest for technological and biomedical applications. Full hydrogenation on both sides of graphene leads to graphane (GH).^1^ Graphane is a 2D material with the same honeycomb structure of graphene, but the change of atomic configuration due to hydrogenation modifies the carbon atom hybridization from sp^2^ into sp^3^, giving rise to a band gap that GW calculations predicted to be in the range 5.4–6.1 eV.^2−5^ Similarly to the effect of hydrogenation, simple arrangements of hydroxyl groups (OH) on graphene change the electronic and optical properties of the pristine material. The synthesis of hydroxylated graphane (HyGH), that is, fully functionalized graphene derivatives with −H, and −OH groups bonded to the carbon atom network in full sp^3^ hybridization, and graphol (highly hydroxylated graphene) was achieved through hydroboration of graphene oxide (GO) followed by protonation.^6^ These compounds showed catalytic properties toward the oxidation of biomarkers and even in hydrogen evolution reactions. Large scale (kilogram scale) synthesis of hydroxylated graphene has been reported in ref (7) through hydrolysis reaction cycles of halogenated graphene.

Ab initio calculations suggest that HyGH possesses the potential to conduct protons in the complete absence of water.^8^ The thermodynamic stability of HyGH structures has been recently theoretically investigated in ref (9). The HyGH structures were build up by progressive substitution of −H with −OH groups and the most stable configuration was selected for each substitution. The formation of HyGH from H_2_ and O_2_ is an exothermic reaction and can be more favorable than the formation of water in the presence of graphene. Moreover, the stability regions in the phase diagram of the most stable structures have been calculated, proving that HyGH structures with low contents of hydrogen are formed for high O partial pressure, while graphane and HyGH structures with high contents of hydrogen functionalization are formed for high H partial pressure.^9^ Several theoretical studies have investigated electronic properties of graphene functionalized with oxygen ad hydroxyl groups,^10−12^ but a systematic analysis of the electronic properties of graphane modified through different hydroxyl substitutions is still missing.

In the present work, we investigate the modification of the electronic properties of stable hydroxylated graphane structures. The electronic band structure, the density of states (DOS), and the electronic affinity (EA) are calculated for each geometry, as a function of the level of hydroxylation. Finally, we show that it is possible to tune the work function (WF) of graphene by forming heterostructures with HyGH. The work is organized as follows: Section 2 describes the theoretical method based on first-principles approach and introduces the models. Section 3 presents the results of the simulation on HyGH related to three subjects: (i) the electronic band structure and DOS for each stage of hydroxylation of graphane and of graphene oxides; (ii) the EA of the studied HyGH structures;and (iii) the WF of the graphene/HyGH heterostructure. In Section 4, we discuss the results in the framework of prospective electronic applications. Finally, the conclusions and the potential impact of HyGH in technology are highlighted in Section 5.

## 2 Methods

The computational approach was based on a pseudopotential plane-wave method using the PWSCF code as implemented in the QUANTUM-ESPRESSO package.^13^ We used the generalized gradient approximation (GGA) with the Perdew, Burke, and Ernzerhof (PBE) exchange-correlation functional.^14^ The pseudopotential plane-wave calculations were performed using Vanderbilt ultrasoft pseudopotentials.^15^ It is well-known that calculations based on GGA functional perform well in the evaluation of structural properties, but they underestimate the energy bandgap. Therefore, we also used the more computationally expensive screened hybrid functional of Heyd, Scuseria, and Ernzerhof (HSE)^16^ which mixes the Hartree–Fock (HF) exchange with the GGA exchange and correlation in the short-range portion of the potential. Indeed, the HSE hybrid functional has been demonstrated to successfully predict the electronic properties of graphene derivatives.^17^ We have calculated the HSE total energy using the default value of 0.25 for the fraction of exact exchange (EXX) and then used a value of EXX equal to 0.50 such that the resulting electronic band gap of graphane was 5.4 eV, as predicted by more accurate GW calculations.^5^ We indicate this latter functional with the name HSE_2_.

All geometry optimizations were performed using the PBE exchange-correlation functional with an energy cutoff for the wave functions of 60 Ry, a cutoff for the charge density of 600 Ry, and 12 × 12 × 1 k-points Monkhorst–Pack grid. The systems were fully relaxed with a convergence threshold of 0.001 Ry/Å on the interatomic forces and imposing a final stress less than 0.04 GPa. We verified that the chosen cut-offs for the wave functions and charge density allow a convergence of the total energy better than 0.002 eV/atom. In all of the examined structures, we have added O and/or H atoms to the hexagonal 2 × 2 unit cell of the graphene, which contains eight carbon atoms. The distance between each monolayer and its periodic image was set to 20 Å. Moreover, in the graphene/HyGH heterostructure calculations, we used the semiempirical Grimme’s DFT-D3 correction^18^ to take into account dispersion forces.

The HyGH structures here considered are indicated with the symbol GH(*n*_OH_OH), where *n*_OH_ hydroxide groups (OH) substitute the same number of hydrogen atoms in the ideal graphane (GH) supercell. The HyGH structures differ for the percentage of hydroxylated C atoms, varying from 12.5% for GH(1OH) to 100%, for GH(8OH). The GH(*n*_OH_OH) structures have all C atoms sp^3^ hybridized as in graphane.

## 3 Results

In the present work, we focus on the electronic properties of the low energy geometries of HyGH^9^ to explore the potential applications in nanotechnology. Moreover, we investigate the use as interfacial layer in 2D material-based heterojunctions.

### Electronic Structure

**Figure 1:**
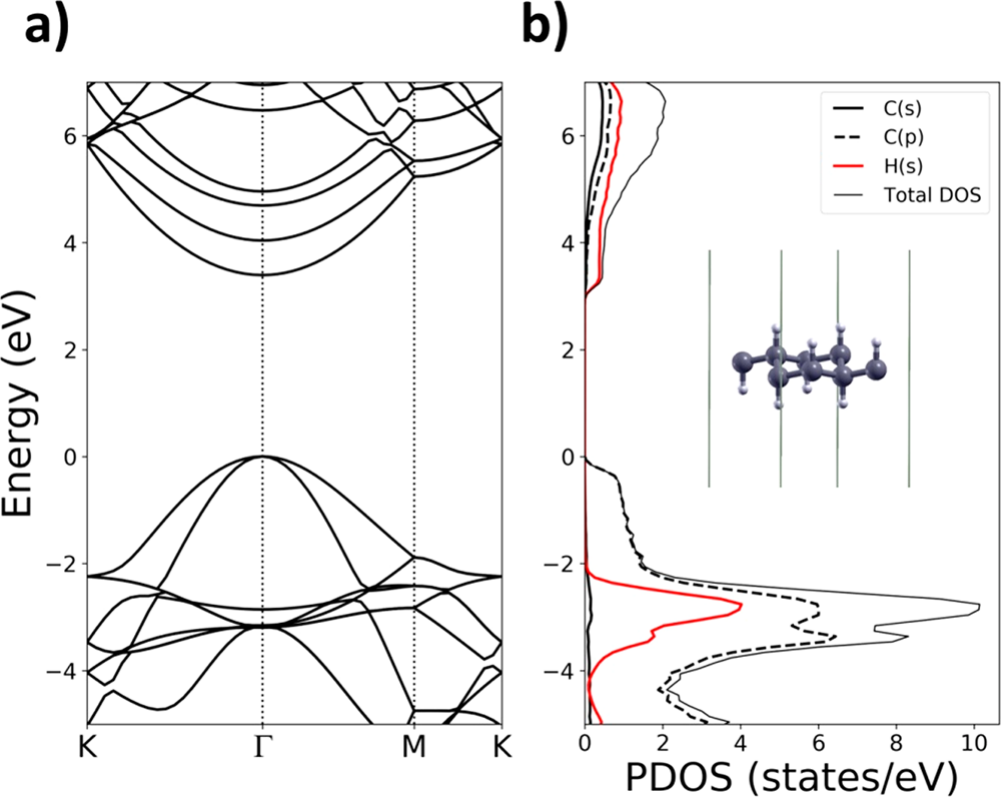
DFT-PBE calculations for graphane: (a) band structure and (b) density of states. In the inset of the panel b, the relaxed geometry of graphane is shown. The top of the valence bands is set to 0 eV. C and H atoms are gray and white, respectively.

The band structure and the projected density of states (PDOS) of pristine graphane are shown in Figure 1, to give a reference for comparison with HyGH. In pristine graphane, we find a direct gap at Γ, with a valence band of C(p) character, and H(s) states strongly contributing to the conduction bands.

**Figure 2:**
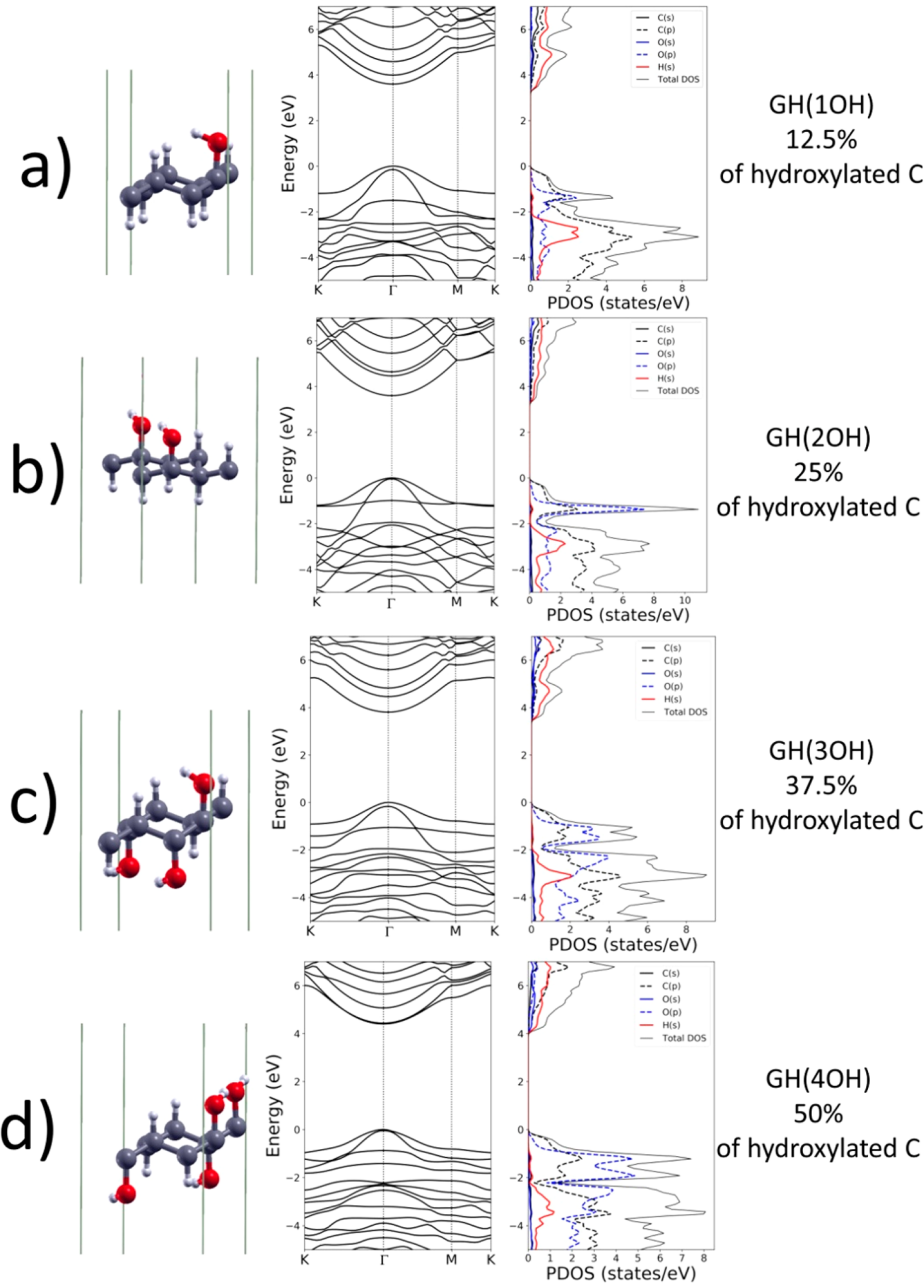
DFT-PBE relaxed geometry, band structure, and density of states of (a) GH(1OH), (b) GH(2OH), (c) GH(3OH), and (d) GH(4OH) hydroxylated graphane. The top of the valence band is set to 0 eV. C, O, and H atoms are gray, red, and white, respectively.

**Figure 3:**
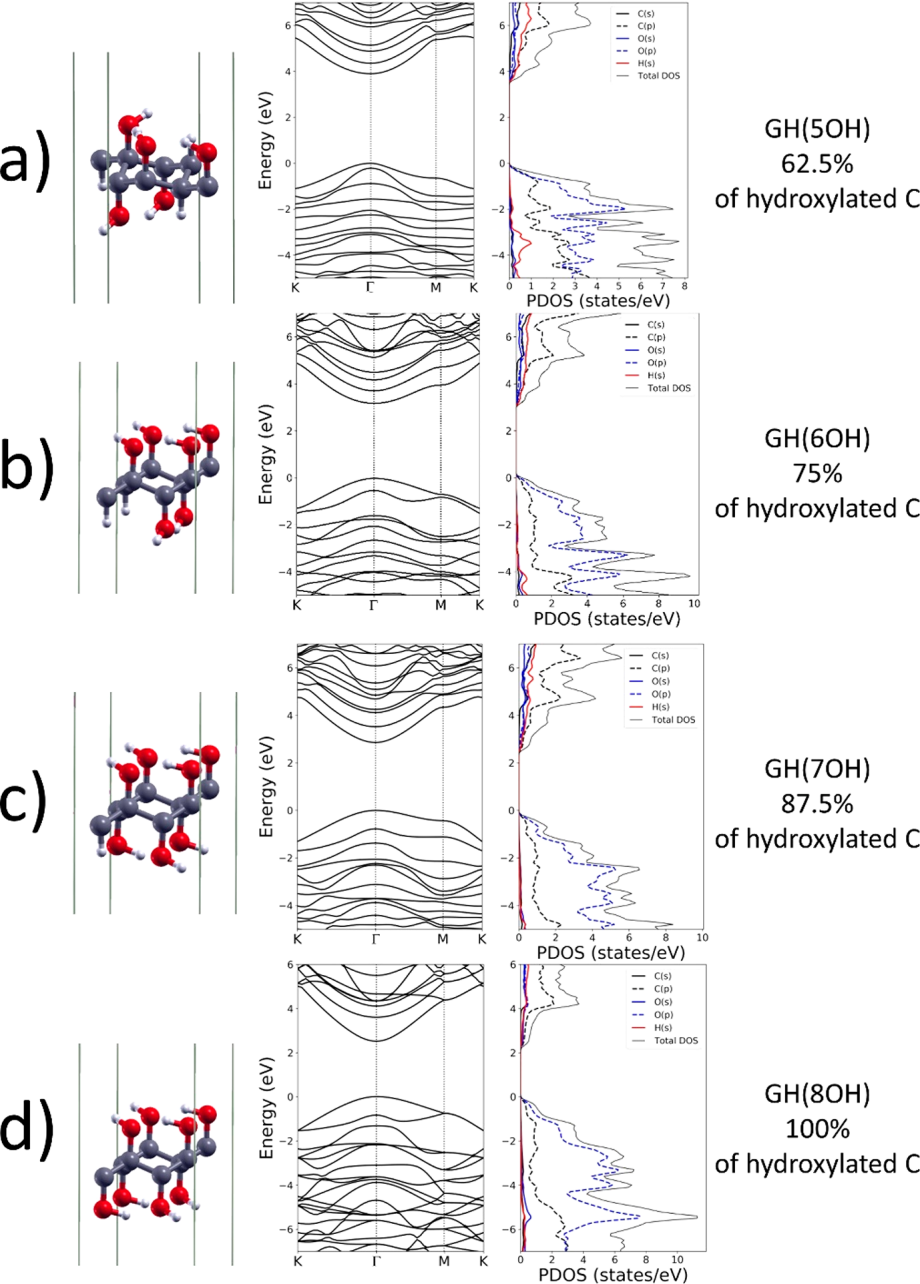
DFT-PBE relaxed geometry, band structure, and density of states of (a) GH(5OH), (b) GH(6OH), (c) GH(7OH), and (d) GH(8OH) hydroxylated graphane. The top of the valence bands is set to 0 eV. C, O, and H atoms are gray, red and white, respectively.

By progressive hydroxylation, the electronic band structures become more and more complex, as shown in Figure 2 and 3. The maximum of valence band (MVB) and the minimum of conduction band (MCB) remain at the Γ high symmetry point for all the investigated structures, hence, the electronic band gap is always direct. By examining the energy bandgaps at the K and M high symmetry points using PBE, we found that these bandgaps reach their highest value for graphane (8.08 and 7.12 eV, respectively, see Table 1). Among all the hydroxylated structures, GH(4OH) has the highest energy gap in K (M) with 7.0 (6.3) eV. The hydroxylation of graphane makes the orbitals of C and H to hybridize with those of oxygen so that new bands appear below and above the gap. The valence band of GH(1OH), compared to the graphane one, is narrowed as a consequence of the resonance of C(p) and O(p) states, confirmed by the overlap of PDOS peaks associated with those states. Therefore, a low dispersion band arises around −2 eV.

**Table 1:** PBE, HSE, and HSE_2_ Fundamental Band Gaps at Γ and PBE Secondary Bandgaps in M and K High Symmetry Points versus Number of OH *n*_OH_ and Percentage of Hydroxylated C in Graphane^a^

*n*_OH_	%	PBE Γ	HSE Γ	HSE_2_ Γ	PBE K	PBE M
0	0	3.4	4.4	5.4	8.1	7.1
1	12.5	3.6	4.7	5.7	6.5	6.1
2	25	3.6	4.7	5.7	6.7	6.2
3	37.5	3.8	5.0	6.1	6.1	5.9
4	50	4.4	5.6	6.7	7.0	6.3
5	62.5	3.9	5.2	6.4	6.6	6.0
6	75	3.1	4.9	5.8	6.1	4.6
7	87.5	2.8	4.4	5.7	6.1	4.8
8	100	2.5	4.1	5.5	6.5	5.1

a The energy units are eV.

The PDOS of GH(2OH) has a large peak at −1.3 eV related to O(p) states overlapping to a less intense peak associated with C(p). The large peak is due to the oxygen-related flat band. We observe that along the M-K path the highest valence band is flat until *n*_OH_ ≤ 4 and becomes more dispersive for *n*_OH_ > 4. By further increasing the hydroxylation, the number of PDOS peaks associated with O(p) states increases and the bands become denser. For example, the PDOS of GH(8OH) shows a dense sequence of peaks related to O(p) states which are more intense than those associated with C(p) states. Indeed, the structure of valence bands becomes quite complex, with several intersecting bands. At the same time, the presence of O(s) and O(p) states in the lowest conduction bands starts to be evident for *n*_OH_ ≥ 4. However, the intensity of C(p) and H(s) PDOS is higher. In most of the hydroxylated structures, the degeneration at M is removed with the exception of GH(2OH) and GH(8OH).

**Figure 4:**
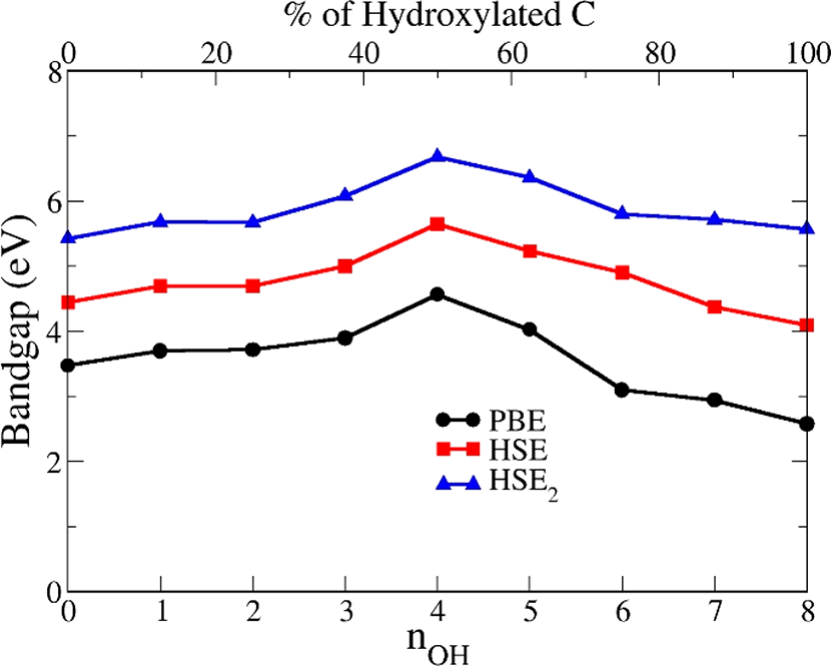
Fundamental electronic band gap at Γ of hydroxylated graphane versus the number *n*_OH_ of OH groups in the supercell and versus the percentage of hydroxylated graphane.

As summarized in Figure 4, by progressive hydroxylation of graphane the fundamental band gap at Γ increases from 5.40 eV (graphane, 0OH, HSE_2_) to 6.68 eV for GH(4OH). When more than 50% of hydrogen atoms are substituted with OH, the gap starts decreasing, reaching its minimum value of 5.57 eV for GH(8OH), where graphene is fully hydroxylated. A qualitatively similar behavior is also found when using other XC functionals, HSE and PBE. This trend of the fundamental gap is due to the interplay between oxygen p states, which create new occupied flat bands, and the change in character of the conduction states, which become less H related.

### Electronic Affinity

**Figure 5:**
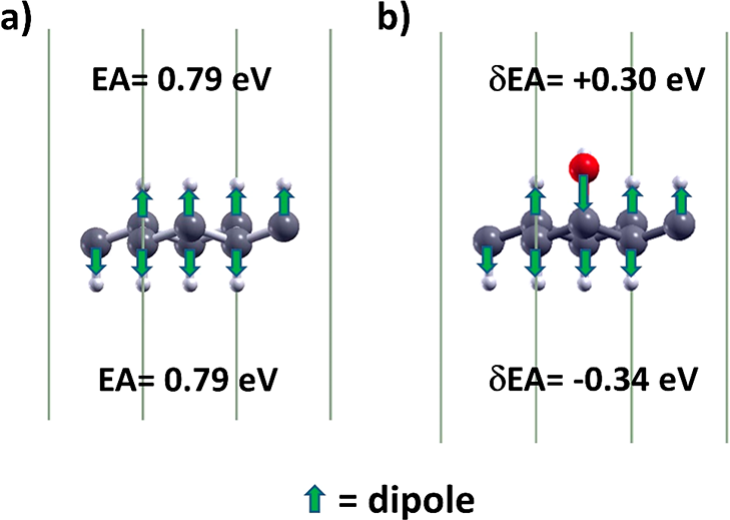
Induced microscopic electric dipoles of (a) graphane and (b) GH(1OH) hydroxylated graphane. HSE_2_ calculated electronic affinity EA and its variation δEA due to the first hydroxylation are shown in the panels (a) and (b), respectively.

In a material, the EA is calculated as EA = *E*_vac_ – *E*_MCB_, where the vacuum energy *E*_vac_ is defined as the electrostatic potential energy in the vacuum, far away from the system, and *E*_MCB_ is the energy of the bottom of the conduction band. The EA can be modified by the presence of different adsorbates on the surface. The EA of graphane calculated within the HSE_2_ functional results to be 0.79 eV, to be compared with the more accurate GW value of 0.4 eV.^19^ Discrepancies between GW and DFT calculations, even when using hybrid functional, are expected. PBE and HSE functionals give EA values of 1.35 and 1.02 eV, respectively, further decreasing the agreement with the GW value. Therefore, in the following we will discuss the EA referring to HSE_2_ results. When hydrogen is adsorbed on graphene, outward-pointing surface dipoles are produced because hydrogen is less electronegative with respect to C. For example, in Figure 5a the induced dipoles along the C–H bonds of graphane are represented. On the other hand, since O is more electronegative than C, an inward-pointing surface dipole is produced that leads to the increase of the EA. For example, in Figure 5b the dipole produced by substitution of −H with −OH is pointing in the opposite direction than the dipoles along C–H bonds on the top vacuum region. This causes the increase of the EA from 0.79 eV (pristine graphane) to 1.09 eV. On the other hand, the dipole along C–O bond is in the same versus of the dipoles along C–H bonds on the bottom vacuum region, so that the EA decreases to 0.45 eV. Therefore, the EA modulation of graphane due to functionalization is more complex than the EA behavior of the functionalized surface of a semi-infinite crystal. Indeed, the EA modification of the latter depends on the dipole orientation induced by atoms adsorption at just one interface with vacuum, while graphane has two such interfaces and the substitution of H atoms on one side influences the EA on both sides. Similar to the case of silicon graphane, the “upper” and “lower” electron affinity in these systems can be different.^20^

**Figure 6:**
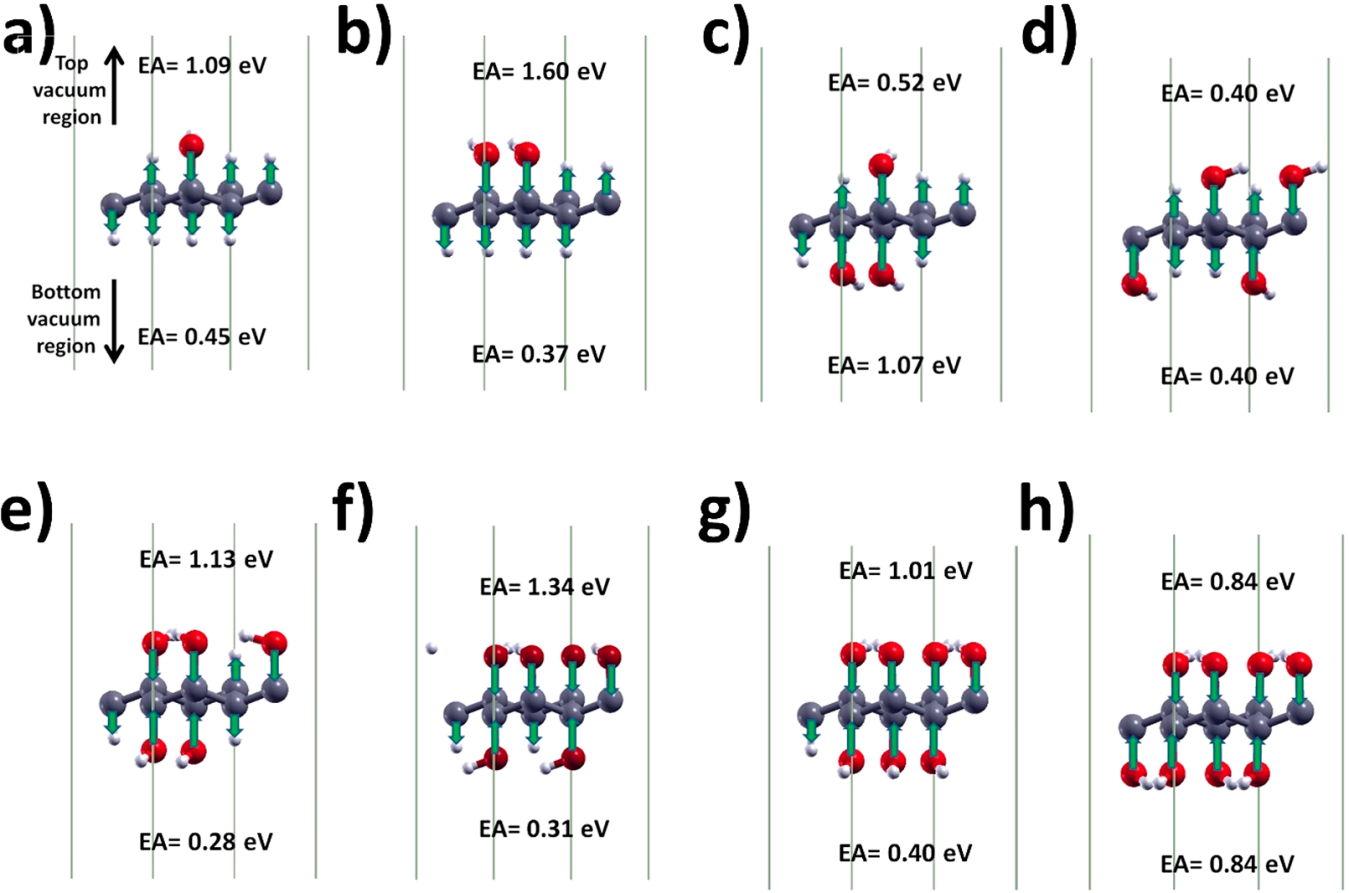
Electronic affinity and induced microscopic electric dipoles of (a) GH(1OH), (b) GH(2OH), (c) GH(3OH), (d) GH(4OH), (e) GH(5OH), (f) GH(6OH), (g) GH(7OH), and (h) GH(8OH) hydroxylated graphane calculated with HSE_2_.

The EA of the up and down symmetric structures GH(4OH) and GH(8OH) is 0.40 and 0.84 eV as shown in Figure 6d,h, respectively. Instead, the EA of the remaining nonsymmetric structures is different on the two sides of each structure. The lowest EA (0.28 eV) is found for the GH(5OH) on the bottom-side of the carbon lattice shown in Figure 6e. Indeed, for GH(5OH) the three inward-pointing microscopic electric dipoles along C–O on the top region add to the two outward-pointing microscopic electric dipoles along C–H on the bottom in such a way to lower the EA. On the other side, for GH(2OH) there are six microscopic electric dipoles on both sides hindering the escape of electrons on the top vacuum region so that the EA reaches the maximum value of 1.60 eV, as shown in Figure 6b. These results will be further discussed in the next section.

### Work Function of the Graphene/GH(1OH) Heterostructure

**Figure 7:**
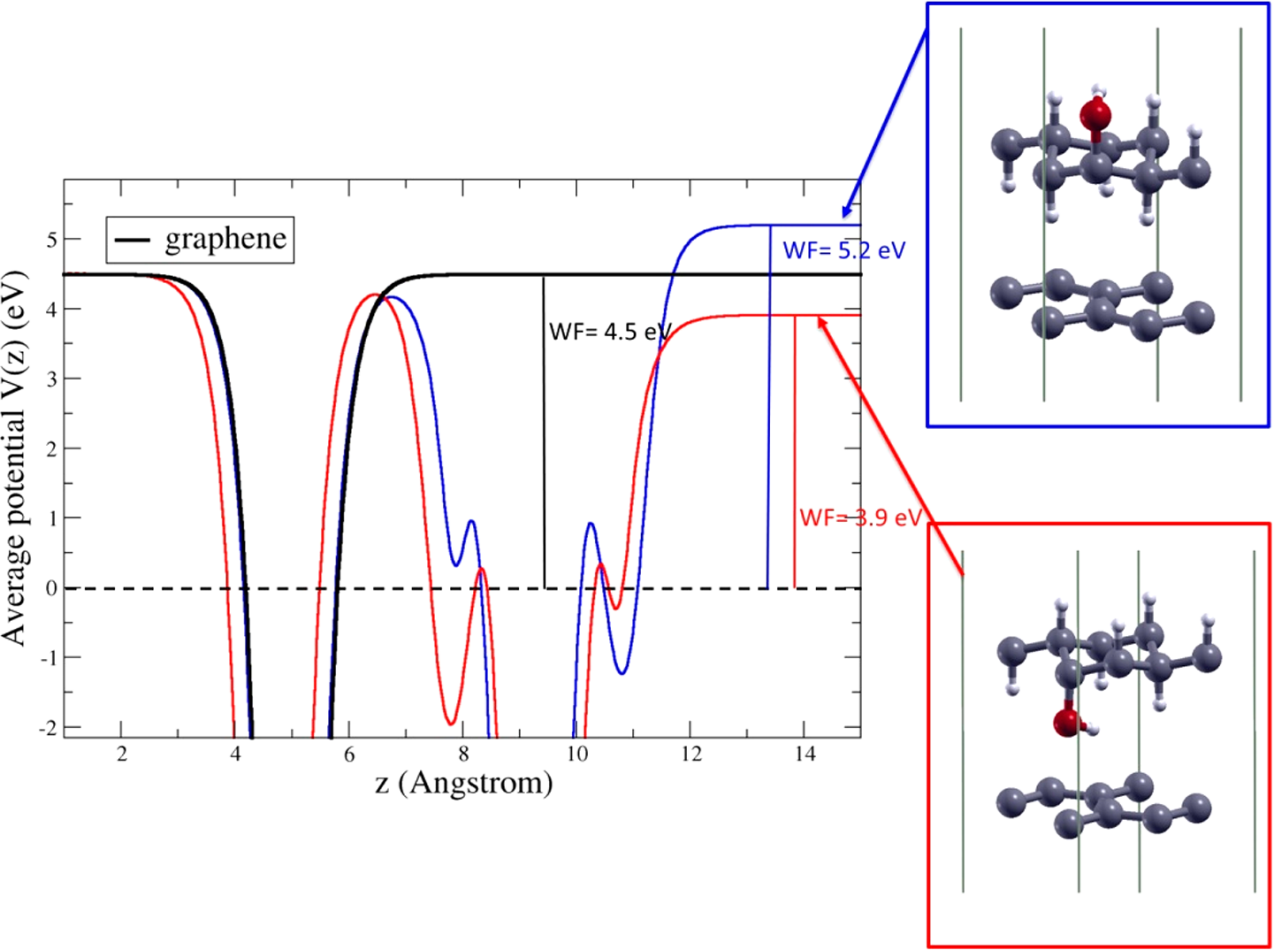
Average electrostatic potential V(z) for pristine graphene (black) and two graphene/GH(1OH) heterostructures (blue and red). The horizontal dashed line indicates the Fermi energy.

As already mentioned, the difference of the “upper” and “lower” EA in the HyGH structures with asymmetric distribution of top and bottom hydroxyl groups is related to the formation of an intrinsic dipole perpendicular to the xy plane. The differences in EA, ΔEA, and the total intrinsic dipole moments, μ_*z*_, are reported in Table 2. Such monolayers can then be used to shift the barrier height of any interface, depending on the orientation of the dipole layer. In this way the WF, given by WF = *E*_vac_ – *E*_F_ can be modified, where *E*_F_ is the Fermi energy. For example, it is possible to tune the WF of graphene by forming heterostructure with HyGH. In Figure 7, the electrostatic potentials (averaged over planes parallel to the sheet) versus the distance *z* (parallel to the sheet normal) are shown for graphene and two graphene/GH(1OH) heterostructures. The heterostructures differ for the orientation of the HyGH intrinsic dipole. The flat potential regions on the right of Figure 7 give the vacuum levels for the different structures. For isolated graphene, we find a WF of 4.5 eV in good agreement with experiments.^21^ When the heterostructures are formed, the distance of the bottom Hydrogen atoms of C–H from the graphene sheet is 2.9 (3.37) Å when the O–H is on the top (bottom) of GH(1OH). The vacuum level is shifted downward of 0.6 eV when the OH group is at the bottom of graphane, resulting in a WF of 3.9 eV; in the other case, when the OH is on top of graphane, the vacuum level is shifted upward of 0.7 eV resulting in a WF of 5.2 eV. The dipole potential of the isolated GH(1OH) (0.64 eV, see Figure 6b and Table 2) is modified by the electron charge redistribution due to the interaction with graphene.

**Table 2:** Difference in EA and Total Intrinsic Dipole Moment μ_*z*_ for the HyGH Structures^a^

	Δ*n*_OH_	|ΔEA (eV)|	|μ_*z*_(D)|
GH(1OH)	1	0.64	0.219
GH(2OH)	2	1.23	0.423
GH(3OH)	1	0.55	0.189
GH(4OH)	0	0.00	0.000
GH(5OH)	1	0.85	0.297
GH(6OH)	2	1.03	0.364
GH(7OH)	1	0.61	0.218
GH(8OH)	0	0.00	0.000

a Δn_OH_ is the difference of the number of OH groups above and below graphane.

## 4 Discussion

Our results show that the sp^3^ hybridization of carbon atoms in graphane and HyGH tunes the energy band gaps of HyGH. The direct energy gap, evaluated using the HSE_2_ functional, starts from 5.4 eV in graphane and then by progressive hydroxylation reaches the maximum value of 6.68 eV on GH(4OH), that is, for the 50% hydroxylated graphane structure. Next, the gap decreases to 5.57 eV on G_O_H(8OH), where graphene is fully hydroxylated. Through PDOS analysis we find that the orbitals of C and H hybridize with those of oxygen, so that new bands appear below and above Fermi energy. Although graphane has a smaller fundamental band gap, it has larger energy gaps at K and M high symmetry points. The calculated energy gaps imply electrical insulation and the presence of an optical absorption edge at low wavelengths, in contrast with graphene which is semimetal with high electronic conductivity and low energy optical absorption edge (zero gap when isolated from the environment) due to the sp^2^ hybridization which frees mobile π-orbital electrons. Therefore, HyGH appears as an extremely interesting novel 2D material with in-plane insulating electrical properties.

We have calculated the EA of the different HyGH structures. The EA depends on the specificity of the surface geometry and on the presence of different adsorbates. In particular, EA variations due to the presence of adsorbates can be assessed very intuitively by comparing the electronegativity of the adsorbate atoms with respect to the substrate ones. In the case of hydrogenated diamond surface, the electron affinity becomes negative^22^ since hydrogen is less electronegative than C. An outward-pointing surface dipole is produced leading to the EA decrease and consequently a reduced local vacuum level.^23^ The same mechanism occurs in graphane. When hydrogen is substituted by hydroxyl groups, inward-pointing surface dipoles are produced that lead to a EA increase on one side and a EA decrease on the opposite side. We found that HyGH EA can be reduced to 0.28 eV, allowing for low-EA 2D materials. However, the hydroxylation process induces asymmetric EA changes because graphane has two interfaces with vacuum, and thus the EA can be different on the two structure sides. This difference results from the formation of an intrinsic dipole through the monolayer, making GH(*n*_OH_OH) a 2D ferroelectric material. The different orientations of the OH in the GH(1OH) allow to decrease or increase the WF of graphene of about 0.6–0.7 eV.

Having a commensurate lattice structure to graphene and affording the WF tunability of different isomers, HyGH family materials could be ideal interlayers in graphene-based devices and 2D heterostructures. Transparent HyGH structures could be designed to serve as interfacial or electron/hole transport layer (ETL/HTL) in optoelectronic devices, such as OLED and solar cells, matching the interfacial energy levels of different materials.^24,25^

## 5 Conclusions

In conclusion, we investigated the modifications of the electronic bands structure, the DOS and the EA of graphane as a consequence of progressive substitution of −H with −OH groups. The C–C sp^3^ bonds determine the large energy gaps of HyGH. The EA is in the range 0.28–1.60 eV depending on the geometric configuration. A finite dipole may appear making GH(*n*_OH_OH) a 2D ferroelectric material. Together with the already known possible applications of HyGH as novel biomaterial to be used in bone and skin regeneration, we envisage the possibility to use HyGH as 2D material with low EA. Moreover, by exploiting the intrinsic dipole we found in some of the structures, we demonstrate that the WF of graphene can be easily tuned by building GH(1OH)/graphene heterostructures. Finally, heterostructures fabricated with HyGH monolayers may play the role of electron transport layer or hole transport layer in solar cells, depending on the polarity of the 2D crystal.

## References

[ref1] Sofo J. O.; Chaudhari A. S.; Barber G. D. Graphane: a Two-Dimensional Hydrocarbon. Phys. Rev. B: Condens. Matter Mater. Phys. 2007, 75 (15), 153401 10.1103/PhysRevB.75.153401.

[ref2] Lebegue S.; Klintenberg M.; Eriksson O.; Katsnelson M. I. Accurate electronic band gap of pure and functionalized graphane from GW calculations. Phys. Rev. B: Condens. Matter Mater. Phys. 2009, 79 (24), 245117 10.1103/PhysRevB.79.245117.

[ref3] Cudazzo P.; Attaccalite C.; Tokatly I. V.; Rubio A. Strong Charge-Transfer Excitonic Effects and the Bose–Einstein Exciton Condensate in Graphane. Phys. Rev. Lett. 2010, 104 (22), 226804 10.1103/PhysRevLett.104.226804. 20867194

[ref4] Pulci O.; Gori P.; Marsili M.; Garbuio V.; Seitsonen A. P.; Bechstedt F.; Cricenti A.; Del Sole R. Electronic and optical properties of group IV two□dimensional materials. Phys. Status Solidi A 2010, 207, 291–299. 10.1002/pssa.200982503.

[ref5] Pulci O.; Gori P.; Marsili M.; Garbuio V.; Del Sole R.; Bechstedt F. Strong excitons in novel two-dimensional crystals: Silicane and germanane. EPL. 2012, 98, 37004 10.1209/0295-5075/98/37004.

[ref6] Poh H. L.; Sofer Z.; Šimek P.; Tomandl I.; Pumera M. Hydroboration of Graphene Oxide: Towards Stoichiometric Graphol and Hydroxygraphane. Chem. - Eur. J. 2015, 21, 8130–8136. 10.1002/chem.201406168. 25877897

[ref7] Sun J.; Deng Y.; Li J.; Wang G.; He P.; Tian S.; Bu X.; Di Z.; Yang S.; Ding G.; Xie X. A New Graphene Derivative: Hydroxylated Graphene with Excellent Biocompatibility. ACS Appl. Mater. Interfaces 2016, 8, 10226 10.1021/acsami.6b02032. 27052945

[ref8] Bagusetty A.; Johnson J. K. Unraveling Anhydrous Proton Conduction in Hydroxygraphane. J. Phys. Chem. Lett. 2019, 10, 518–523. 10.1021/acs.jpclett.8b03627. 30649884

[ref9] Buonocore F.; Capasso A.; Lisi N. An ab initio study of hydroxylated graphane. J. Chem. Phys. 2017, 147, 104705 10.1063/1.4986858. 28915759

[ref10] Boukhvalov W.; Katsnelson M. I. Modeling of Graphite Oxide. J. Am. Chem. Soc. 2008, 130, 10697–10701. 10.1021/ja8021686. 18627149

[ref11] Lahaye R. J. W. E.; Jeong H. K.; Park C. Y.; Lee Y. H. Density functional theory study of graphite oxide for different oxidation levels. Phys. Rev. B: Condens. Matter Mater. Phys. 2009, 79, 125435 10.1103/PhysRevB.79.125435.

[ref12] Yan J. A.; Xian L.; Chou M. Y. Structural and Electronic Properties of Oxidized Graphene. Phys. Rev. Lett. 2009, 103, 086802 10.1103/PhysRevLett.103.086802. 19792747

[ref13] Giannozzi P.; Baroni S.; Bonini N.; Calandra M.; Car R.; Cavazzoni C.; Ceresoli D.; Chiarotti G. L; Cococcioni M.; Dabo I.; Dal Corso A.; de Gironcoli S.; Fabris S.; Fratesi G.; Gebauer R.; Gerstmann U.; Gougoussis C.; Kokalj A.; Lazzeri M.; Martin-Samos L.; Marzari N.; Mauri F.; Mazzarello R.; Paolini S.; Pasquarello A.; Paulatto L.; Sbraccia C.; Scandolo S.; Sclauzero G.; Seitsonen A. P; Smogunov A.; Umari P.; Wentzcovitch R. M QUANTUM ESPRESSO: A Modular and Open-Source Software Project for Quantum Simulations of Materials. J. Phys.: Condens. Matter 2009, 21, 395502 10.1088/0953-8984/21/39/395502. 21832390

[ref14] Perdew J. P.; Burke K.; Ernzerhof M. Generalized Gradient Approximation Made Simple. Phys. Rev. Lett. 1996, 77, 3865–3868. 10.1103/PhysRevLett.77.3865. 10062328

[ref15] Vanderbilt D. Soft Self-Consistent Pseudopotentials in a Generalized Eigenvalue Formalism. Phys. Rev. B: Condens. Matter Mater. Phys. 1990, 41, 7892 10.1103/PhysRevB.41.7892. 9993096

[ref16] Heyd J.; Scuseria G. E.; Ernzerhof M. Hybrid functionals based on a screened Coulomb potential. J. Chem. Phys. 2003, 118, 8207–8215. 10.1063/1.1564060.

[ref17] Barone V.; Hod O.; Peralta J. E.; Scuseria G. E. Accurate Prediction of the Electronic Properties of Low-Dimensional Graphene Derivatives Using a Screened Hybrid Density Functional. Acc. Chem. Res. 2011, 44, 269–279. 10.1021/ar100137c. 21388164

[ref18] Grimme S.; Antony J.; Ehrlich S.; Krieg H. A consistent and accurate ab initio parametrization of density functional dispersion correction (DFT-D) for the 94 elements H-Pu. J. Chem. Phys. 2010, 132, 154104 10.1063/1.3382344. 20423165

[ref19] Marsili M.; Pulci O. The fascinating physics of carbon surfaces: first-principles study of hydrogen on C(0 0 1), C(1 1 1) and graphene. J. Phys. D: Appl. Phys. 2010, 43, 374016 10.1088/0022-3727/43/37/374016.

[ref20] Gori P.; Pulci O.; Marsili M.; Bechstedt F. Side-dependent electron escape from graphene- and graphane-like SiC layers. Appl. Phys. Lett. 2012, 100, 043110 10.1063/1.3679175.

[ref21] Yu Y. J.; Zhao Y.; Ryu S.; Brus L. E.; Kim K. S.; Kim P. Tuning the graphene work function by electric field effect. Nano Lett. 2009, 9, 3430–3434. 10.1021/nl901572a. 19719145

[ref22] Himpsel F. J.; Knapp J. A.; VanVechten J. A.; Eastman D. E. Quantum photoyield of diamond(111)-A stable negative-affinity emitter. Phys. Rev. B: Condens. Matter Mater. Phys. 1979, 20, 624 10.1103/PhysRevB.20.624.

[ref23] Cahen D.; Kahn A. Electron Energetics at Surfaces and Interfaces: Concepts and Experiments. Adv. Mater. 2003, 15, 271–277. 10.1002/adma.200390065.

[ref24] Husain A. A. F.; Hasan W. Z. W.; Shafie S.; Hamidon M. N.; Pandey S. S. A review of transparent solar photovoltaic technologies. Renewable Sustainable Energy Rev. 2018, 94, 779–791. 10.1016/j.rser.2018.06.031.

[ref25] Mustonen P.; Mackenzie D. M. A.; Lipsanen H. Review of fabrication methods of large-area transparent graphene electrodes for industry. Front. Optoelectron. 2020, 13, 91–113. 10.1007/s12200-020-1011-5. PMC7362318 36641556

